# Environmental and genetic interactions in autism spectrum disorder: a literature review with a focus on air pollution

**DOI:** 10.1186/s12940-026-01321-8

**Published:** 2026-07-31

**Authors:** Muhammad Ayyan, Huda Salameh, Sushanth Alladaboina, Sama Ayoub, Abdulla Subai, Hassan Al Naimi, Anfal Sher Khan, Mahmoud Yousef, Mujtaba Pal, Ali Chaari

**Affiliations:** 1 Weill Cornell Medical College Qatar, Doha, Qatar; 2 Department of Biochemistry, Premedical Division, Weill Cornell Medicine Doha, Doha, Qatar

**Keywords:** Autism Spectrum Disorder (ASD), Neurodevelopmental Disorders, Air Pollution, Genetic Factors, Oxidative Stress

## Abstract

Autism Spectrum Disorder (ASD) is a neurodevelopmental condition which is associated with complex interactions between genetic susceptibility and environmental exposures. More recently, air pollution has been investigated as a modifiable risk factor and contributor to the neurodevelopmental outcomes of the disorder. This review focuses on the Middle East and North Africa (MENA) region and discusses genetic, immunologic, and epigenetic findings that help explain how pollutants, including particulate matter (PM₂.₅, PM₁₀), nitrogen oxides, ozone, and dioxins, contribute to the pathogenesis of ASD and its specific involvement in neuronal pathways. Many pollutants have been found to increase oxidative stress, neuroinflammation, and endocrine disruption which alters DNA methylation and histone modification patterns. The pathology of this involves activation of the aryl hydrocarbon receptor (AhR) and its downstream CYP1A1/CYP1B1 pathways which link environmental to altered gene expression and neural circuits. Current studies have shown that prenatal and early postnatal exposures are critical windows in which air pollutants can impair synaptogenesis, microglial regulation, and neurotransmitter balance. Significantly, sex-specific differences further modulate susceptibility, with males demonstrating heightened vulnerability due to hormonal and immune interactions. While progress has been made in uncovering these mechanisms, challenge in establishing causality and addressing diagnostic biases persists. Future directions should integrate longitudinal cohorts, omics-based biomarkers, and machine-learning models to clarify exposure–response relationships and guide personalized prevention strategies. Understanding how environmental and genetic factors converge to disrupt neurodevelopment is essential to mitigating ASD risk in increasingly polluted environments.

## Introduction

Autism spectrum disorder (ASD) is a neurodevelopmental disorder characterized by impairments in communication, deficits in social interactions, and restrictive, repetitive behaviors [50]. Ever since the formal recognition in the year 1943 as a disorder on its own, ASD’s prevalence has risen sharply and it has become a major global public health concern. According to the U.S. Centers for Disease Control and Prevention’s Autism and Developmental Disabilities Monitoring (ADDM) Network, the prevalence among 8-year-olds increased from 67 per 10,000 in 2000 to 230 per 10,000 in 2018, and further to approximately 1 in 36 in 2020 [78]. A recent global meta-analysis similarly reported a pooled prevalence of about 1 in 100 children worldwide [115], underscoring the urgent need to elucidate the genetic and environmental factors contributing to this rise to improve early detection, targeted interventions, and preventive strategies.

Even though the exact etiology of ASD is not clear, there is substantial evidence which supports a multifactorial etiology involving both genetic and environmental factors [84]. Most cases of ASD are attributed to polygenic mechanisms with numerous autosomal and X-linked mutations linked to its occurrence. The development of ASD can also be influenced by monogenic disorders such as tuberous sclerosis (TSC1, TSC2), *UBE3A* gene (Angelman syndrome), and fragile X syndrome (FMR1), which highlights the genetic heterogeneity underlying its etiology [40].

Moreover, there has been increasing evidence which suggests that air pollution may contribute to the pathogenesis of ASD. Air pollution poses a significant public health risk, with 92% of the world's population living in areas exceeding the World Health Organization's Air Quality Guidelines for fine particulate matter (PM2.5) [110]. Air pollution has been shown to induce neurotoxicity through oxidative stress, inflammation, and disruption of the blood–brain barrier, mechanisms implicated in ASD pathogenesis [19]. Additionally, exposure to airborne pollutants has been associated with epigenetic changes in the placenta, potentially altering fetal development and increasing the risk of ASD [75]. Alarmingly, projections suggest that emissions of these harmful pollutants could double within the next two decades, underscoring the need for stricter environmental regulations and targeted research to reduce their impact on neurodevelopmental disorders, including ASD [32].

PM2.5 is particularly hazardous due to its small size and volatility which allows it to penetrate into the body, allows it to cross biological barriers, and allows it to remain airborne for extended periods [54]. Additionally, its low sedimentation velocity keeps it suspended in the air for extended periods, increasing the likelihood of inhalation [34]. Similarly, early exposure to NOx, O3, CO, and SO2 has been linked to a higher risk of ASD in children [54]. NOx is primarily released during combustion processes in vehicles and industrial activities [79]. These compounds can react with atmospheric oxygen to produce O3, which is harmful to cognitive development. CO, a colorless, odorless gas, is another byproduct of incomplete fossil fuel combustion and is associated with ASD.

Additionally, SO2, predominantly emitted from the burning of fossil fuels, contributes to air pollution by acting as a respiratory irritant [79]. Its prenatal and early life exposure has been found to increase the risk of ASD [90]. DPM (Diesel Particulate Matter) is a subset of PM2.5 which gets emitted from diesel engines as a combustion byproduct and comprises carbon-based compounds like elemental carbon, organic carbon, and sulfates [72]. Exposure to DPM is linked to oxidative stress and epigenetic modifications, including changes to gene regulatory regions that influence ASD-related genes [13]. Dioxins represent a group of highly toxic and persistent air pollutants that can be categorized into three main groups: Polychlorinated Dibenzo-p-Dioxins (PCDDs), Polychlorinated Dibenzofurans (PCDFs), and Dioxin-Like Polychlorinated Biphenyls (DL-PCBs), each of which carries adverse health risks to humans and ecosystems [46]. Dioxins have been implicated in inducing neurotoxic effects on the developing brain, which interfere with neurotransmitter systems in the brain, cause immunomodulatory effects, and induce epigenetic changes, all of which are suggested to contribute to ASD development [46].

This review provides a comprehensive analysis of the etiology of ASD by integrating genetic, biological, immune, environmental, and nutritional factors. It dives into the mechanisms underlying ASD risk, with a focus on genetic factors, epigenetic modifications, and immune dysregulation and their contributions to neurodevelopmental abnormalities. This review focuses on the effect of air pollution and how it does so through mechanisms such as oxidative stress, inflammation, and disruptions in neurodevelopmental processes. The roles of nutritional elements such as maternal folic acid and vitamin B12 is also discussed with regards to its protective effects against ASD. Additionally, this review has a specific focus on the Middle East and North Africa (MENA) region as it experiences some of the highest air pollution levels in the world and is underrepresented in the ASD enviornment literature.

### Mechanisms underlying the pathogenesis of autism spectrum disorder

#### Genetic factors and heritability

ASD is known to have a strong genetic basis with an estimated heritability of approximately 80% [104]. A recent large-scale exome sequencing study identified 102 genes associated with ASD risk [105]. The associated mutations are primarily rare, de novo, germline mutations that have deleterious effects on protein function [80]. Genetic changes that underlie ASD often tend to involve structural and sequence variations in genomic DNA such as copy number variations (CNVs) and single-nucleotide polymorphisms (SNPs) [12].

CNVs are structural genetic mutations involving tens of thousands to millions of nucleotides, including deletions or duplications that alter gene copy numbers [80]. Initially identified as part of normal human genetic diversity following the completion of the Human Genome Project, large and de novo CNVs have since been strongly associated with ASD [108]. CNVs occur in approximately 6–10% of individuals with ASD. They are frequently related to monogenic cases (although they can also be multigenic) [12], with 50% of de novo CNVs linked to ASD risk [103]. Exome analysis has identified six specific risk loci on chromosomes 1, 3, 7, 15, 16, and 22 that are strongly associated with ASD, including duplications at 15q13 and microdeletions at 16p11.2 [103].

ASD typically results from contributions by multiple genes, but it is important to realize monogenic forms of ASD exist and these involve abnormalities caused by a single gene. These monogenic mutations can arise from either CNVs or SNPs and can be further categorized into syndromic ASD, which co-occur with genetic disorders, and non-syndromic autism, where ASD manifests without additional clinical features [12].

Syndromic forms of ASD include monogenic mutations in the *FMR1* gene (Fragile X syndrome), *TSC1* and *TSC2* genes (tuberous sclerosis), and the *UBE3A* gene (Angelman syndrome). Each syndrome demonstrates unique pathophysiological mechanisms, leading to significant intellectual disabilities, with ASD being a prominent clinical feature [12, 40]. For instance, Fragile X syndrome results from abnormal FMR1 CGG repeats, leading to gene silencing and impaired synaptic function. Tuberous sclerosis results from mutations in TSC1 or TSC2 that deregulate cell growth, leading to tumor-like lesions in the brain and other organs. Angelman syndrome arises from UBE3A mutations, which disrupt ubiquitin-protein ligase activity and impair synaptic transmission, leading to cognitive and developmental deficits [12].

Monogenic mutations, whether syndromic or non-syndromic, frequently affect signaling pathways such as PTEN and mTOR, as well as genes involved in synaptogenesis, leading to impaired synaptic plasticity, a central feature of ASD pathophysiology [12]. Genes encoding synaptic cell-adhesion molecules—including neuroligins, neurexins, contactins, and cadherins—are particularly implicated. De novo copy number variations in *NRXN1-3* genes, for instance, alter synaptic transmission and neural connectivity, producing behavioral phenotypes characteristic of ASD [42, 117].

Twin studies provide evidence for the genetic basis of ASD, consistently demonstrating higher concordance rates in monozygotic twins compared to dizygotic twins. For example, a study in England and Wales estimated ASD heritability between 56 to 95%, with monozygotic twins showing significantly higher trait correlations (0.77–0.99) than dizygotic twins (0.22–0.65) [27]. Similarly, a Danish study reported a concordance rate of 95.2% in monozygotic twins compared to 4.3% in dizygotic twins, further reinforcing the genetic component of ASD [87].

Although ASD has a strong genetic basis, not all individuals carrying pathogenic variants or high-risk alleles develop the disorder, suggesting that environmental exposures may modulate genetic susceptibility. Increasing evidence supports the concept of gene–environment interactions, wherein specific genetic backgrounds heighten vulnerability to environmental insults such as air pollution. For instance, it was found that children with elevated polygenic risk or high copy number variation (CNV) have a greater neurodevelopmental sensitivity to pollutant exposure. Consistent with this, Kim et al. (2017) demonstrated in his study that ozone exposure amplified the risk of ASD among children who had a high CNV burden which further highlights the synergistic effects between genetic predisposition and environmental stressors. This further supports the multifactorial etiology of ASD and conceptually bridges genetic risk with ecological influences on neurodevelopment.

#### Epigenetic factors

Epigenetic modifications caused by environmental and pathogenic factors, including DNA methylation and post-translational histone modifications, are closely associated with the pathogenesis of ASD [38]. These alterations can significantly impact gene expression, contributing to the development of ASD phenotypes. Epigenetic changes linked to ASD have been identified in several genes, including *OXTR*, *SHANK3*, *NLGN3*, *NLGN4*, *AFF2*, *NRXN1*, *AUTS2*, *GABRB3*, *SLC6A4*, *BCL2*, and *UBE3A* [140]. For example, methylation of the *OXTR* promoter inhibits its expression, disrupting its physiological roles in psychosocial stress response, social bonding, and reproductive behaviors [69]. This disruption has been linked to difficulties in forming emotional connections and challenges in recognizing, interpreting, and responding to social cues, all of which are characteristic of ASD [135]. Notably, environmental exposures, such as airborne pollutants and other toxins, can induce epigenetic modifications similar to those caused by genetic mutations. Prenatal exposure to air pollution has been associated with DNA methylation changes at autism-related loci which further suggests that environmental factors may contribute directly to the epigenetic dysregulation observed in ASD. *SHANK3*, essential for synapse formation, undergoes methylation at CpG island 2, with peak levels occurring two weeks postnatally, coinciding with synapse maturation and suggesting a potential role in ASD-related disruptions to synaptic development and plasticity [140].

Histone modifications, including methylation, acetylation, SUMOylation, ubiquitination, and phosphorylation, play a crucial role in regulating gene expression and are significantly implicated in the pathogenesis of ASD [111]. Such modifications then alter chromatin structure and transcriptional activity which influences neurodevelopment and behavior. For example, an experimental study revealed that methylation of H3 histone protein (H3K4) is linked to alterations in social interactions and repetitive behaviors characteristic of ASD [3, 56]. This implies that the methylation of histone can affect genes involved in neurodevelopmental processes and contribute to ASD-associated behavioral phenotypes. Additionally, histone acetylation, particularly in genes encoding oxytocin and vasopressin receptors, is associated with ASD-like behaviors [119]. Acetylation enhances the accessibility of these genes, increasing oxytocin and vasopressin receptor expression and affecting downstream signaling pathways (H. [126]. Evidence shows that histone deacetylase inhibitors promote receptor upregulation, further supporting their involvement in social behaviors [12]. Research on postmortem brains of individuals with ASD has revealed widespread histone acetylation in the cerebral cortex and cerebellum, along with reduced trimethylation of H3K27 [114]. These epigenetic alterations disrupt neuronal function in the prefrontal cortex, a region critical for cognitive and behavioral regulation, contributing to the behavioral and cognitive deficits observed in ASD [57]. Together, these findings highlight the complex role of histone modifications in ASD etiology and suggest potential epigenetic targets for therapeutic interventions. Recent epigenome-wide studies continue to reveal widespread differences in DNA methylation and histone modification patterns in ASD brains, showing that epigenetic dysregulation remains a consistent and reproducible feature of the disorder. Moreover, recent research has linked these epigenetic changes to neuroinflammatory and oxidative stress pathways [121], further bridging molecular and environmental mechanisms underlying ASD pathogenesis.

#### Immune dysregulation

The immune system, which plays a fundamental role in maintaining the body's homeostasis, is intricately connected to body systems and organs. Recent advancements in neuroendocrinology, neuroanatomy, and cell biology have highlighted the complicated bidirectional pathways between the central nervous system and the immune system, elucidating the role of immune regulation in neurodevelopmental processes [31]. Immune dysregulation in ASD can originate prenatally (through maternal immune activation) and continue postnatally (through intrinsic neuroimmune abnormalities).

Accumulating evidence suggests that maternal immune dysregulation, during early fetal development, particularly within the first trimester, increases the risk of neurodevelopmental disorders, including ASD [29]. The inflammatory response associated with maternal immune activation (MIA) may lead to the production of brain-reactive antibodies that can cross the placenta from the mother to the fetus, altering fetal brain structure and function [16]. Further analysis of maternal cytokine profiles and placental immune responses may help identify the specific mechanisms through which MIA contributes to neurodevelopmental disruptions in ASD.

Gene expression studies in ASD have revealed dysregulated immune pathways with postmortem analysis of ASD brain tissues showing upregulation of genes associated with immune and inflammatory responses [37]. Epigenomic investigations have further identified dysregulated microRNA (miRNA) expression in individuals with ASD, impacting ASD-associated genes and influencing immune and metabolic processes [134]. Additionally, genome studies have identifies common gene variants which affect similar immune pathways further linking them to ASD-like traits and increased susceptibility to ASD. One such genetic variation involves the IL-6 gene. Elevated levels of IL-6 levels impairs neuronal adhesion, migration, and synapse development which is responsible for processing critical functions of the brain and may contribute to ASD pathogenesis [37].

**Fig. 1:**
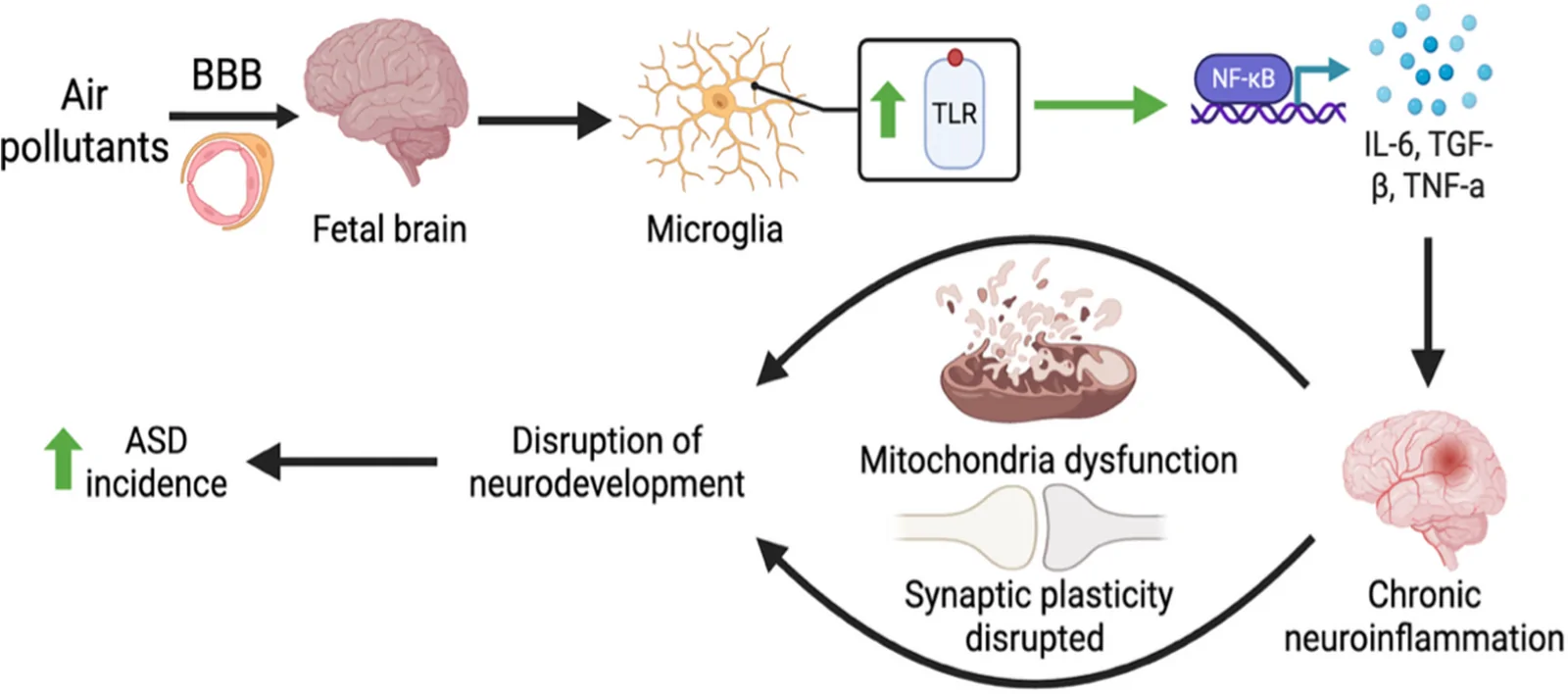
Air pollution-induced neuroinflammation: microglial activation and cytokine dysregulation in ASD pathogenesis

Microglial (the brain’s resident immune cells) activation is another hallmark of immune dysregulation in ASD. Postmortem brain studies have revealed increased production and density of microglia cells across various brain regions implicated in ASD, including the dorsolateral prefrontal cortex, fronto-insular and visual cortex, cerebellum, midfrontal, and cingulate gyrus [85, 118, 123]. This excessive microglial activity is associated with changes in gene expression levels, potentially driven by inflammatory cytokines [123]. Notably, several environmental factors—including air pollution—can serve as potent triggers for these immune responses. Figure 1 illustrates how inhaled pollutants can initiate microglial activation and cytokine release, linking environmental exposure to neuroinflammation in ASD. Figure 1 illustrates this proposed mechanism, showing how air pollutants can cross the blood–brain barrier, activate microglia, and promote the release of pro-inflammatory cytokines (IL-6, TNF-α, TGF-β), ultimately contributing to chronic neuroinflammation, impaired neurodevelopment, and increased ASD incidence.

A transcriptomic study of identical twins with ASD further demonstrated immune dysregulation, identifying differential gene expression in immune-related pathways [101]. Additionally, RNA sequencing in adults with ASD revealed contributions of both innate and adaptive immunity to ASD pathogenesis [53]. These findings align with cytokine profile abnormalities observed in individuals with ASD, including elevated levels of pro-inflammatory cytokines (TNF-α, IL-6, IL-8) and decreased levels of anti-inflammatory cytokines (TGF-β, IL-10) [53].

Gene expression analyses identified 117 significantly upregulated and 83 downregulated genes, with immune dysregulation associated with upregulated genes. Several pathways were affected, including myeloid leukocyte-mediated immunity, neutrophil degranulation, and Th1 and Th2 activation, further suggesting that immune dysregulation plays a crucial role to ASD pathology [53].

Investigations into peripheral immune responses have revealed significant alterations in cytokine profiles among individuals with ASD. Elevated plasma levels of pro-inflammatory cytokines (IL-1β, IL-6, IL-8, IL-12p40) have been identified in children with regressive forms of ASD. These immune disruptions correlate with behavioral impairments, highlighting the correlation between immune dysregulation and ASD symptomatology [10].

#### Nutritional factors

A growing body of research suggests that maternal nutrition may play a role in the etiopathogenesis of ASD, with folic acid and vitamin B12 and vitamin D deficiencies identified as major contributors [20].

A randomized controlled trial revealed a significant inverse association between maternal folic acid intake during early pregnancy and ASD risk in offspring [107]. Specifically, maternal consumption of ≥ 600 μg folic acid daily during the first month of pregnancy was linked with a significant reduction in ASD risk, with an odds ratio of 0.62 (95% CI: 0.42–0.92). Notably, this association was robust among mothers and children with specific MTHFR 677 C > T variant genotypes. The MTHFR 677 C > T genetic polymorphism is linked to elevated homocysteine levels, which increase the individual's folate requirements for normal neurodevelopment, making them susceptible to ASD. Folate and vitamin B12 are important cofactors in the one-carbon metabolism pathway as they support DNA methylation and the expression of gene regulation. Deficiency in these nutrients can impair methylation capacity, potentially exacerbating epigenetic vulnerabilities in neurodevelopment and contributing to ASD risk. This explains why fetuses whose mothers have higher folic acid intake during pregnancy have been observed to exhibit a reduced risk of developing ASD. Emerging evidence also suggests maternal vitamin D deficiency may be associated with elevated ASD risk, though findings are mixed. A Finnish case–control analysis reported ~ 1.4 × higher odds of ASD if the mother’s mid-pregnancy 25(OH)D was deficient (< 30 nmol/L) [112]. In contrast, the UK ALSPAC birth cohort found no significant overall link between the prenatal 25(OH)D and ASD. Prenatal deficiencies of vitamin D could impair fetal brain development as it is a neuroactive hormone that contributes to neuronal differentiation, neurotransmitter synthesis, and cytokine signaling in the developing brain [77].

In addition to maternal nutrition, early childhood diet is also being studied for its links to ASD risk. A randomized controlled trial conducted in China found that children with ASD had significantly lower folate/B12 levels, which correlated with poorer language and adaptive behaviors, suggesting that these nutrients may influence neurodevelopment [134]. In this study, serum folate and vitamin B12 levels were positively correlated with various developmental aspects, particularly language and adaptive behavior, emphasizing the potential impact of these nutrients on neurodevelopment in children with ASD. However, the body of literature on this topic is still emerging and currently lacks sufficient evidence. Some studies have found no substantial correlation between folate serum levels/intake and ASD risk. Other studies have paradoxically found that mothers of autistic children had received high-dose folic acid supplementation during pregnancy, making it difficult to draw definitive conclusions [20]. Additionally, diet-linked factors may operate through the gut–brain axis. Nutrient intake and diet composition are known to directly effect the gut’s microbiome which directly influences neurodevelopment through immune, metabolic, and neurotransmitter pathways.

In addition to vitamins, an early diet may influence ASD risk via the gut microbiome. New research investigating the possible bidirectional role of the microbiota-gut-brain axis in ASD suggests that individuals with ASD have a distinct gut microbiota profile compared to neurotypical individuals [62]. These differences include changes in bacterial diversity and the relative abundance of specific bacterial species, particularly within the genera *Bacteroides*, *Clostridia*, and *Lactobacillus*. An increase in pathogenic Clostridium species and a reduction in beneficial bacteria such as *Bifidobacterium* have been associated with ASD symptoms.

#### AhR, CYP1A1, CYP1B1 pathways

The aryl hydrocarbon receptor (AhR) is a ligand-activated transcription factor within the basic-helix-loop-helix (bHLH)/Per-ARNT-Sim (PAS) family. It plays a critical role in processes such as cell differentiation, proliferation, carcinogenesis, and immune response regulation. Additionally, AhR is integral to several physiological functions, including host defense, immunity, stem cell maintenance, and xenobiotic metabolism [64]. Historically, AhR was believed to be activated exclusively by environmental pollutants such as polycyclic aromatic hydrocarbons (PAHs). However, recent findings have identified non-PAH molecules, including ketoconazole and heavy metals, as modulators of AhR activity, further expanding its biological significance [33].

AhR activation has been implicated in a variety of pathological conditions, including neurodevelopmental disorders such as ASD. Human AhR resides in the cytosol as a complex with the chaperone protein HSP90 and the inhibiting protein XAP2. Upon its activation by ligand binding, AhR undergoes conformational changes, dissociates from HSP90, and translocates to the nucleus. In the nucleus, AhR forms a heterodimer with the AhR nuclear translocator (ARNT), which binds to xenobiotic response elements (XREs) on specific genes, initiating their transcription and translation [120]. This binding activates the transcription of several genes, including *CYP1A1*, *CYP1A2*, and *CYP1B1*. The CYP1 family encodes enzymes that bioactivate environmental toxicans into reactive intermediates, such as epoxides. These intermediates can oxidize macromolecules, such as DNA, leading to DNA adducts and strand breaks, which are associated with neurodevelopmental disruptions [93].

Dioxin-like chemicals, a class of persistent organic pollutants, are potent AhR activators associated with an increased risk of ASD. These chemicals exert harmful effects during pregnancy by activating AhR and its downstream pathways in the placenta, a critical site for fetal development. For example, high AhR expression has been observed in the amniotic fluid, indicating that these chemicals can cross the placenta. Consequently, CYP1A1, CYP1A2, and CYP1B1 genes may be aberrantly activated by environmental toxicans, increasing ASD risk [74]. Animal models provide additional evidence of AhR’s role in ASD pathogenesis. In pregnant mice, constitutively activated AhR signaling disrupted neuronal migration during hippocampal development, leading to altered brain structure and behavior in their offspring. This suggests that AhR activation during critical developmental windows can have lasting neurodevelopmental consequences, potentially contributing to ASD phenotypes [68].

Acetylcholinesterase (AChE) is an enzyme responsible for hydrolyzing acetylcholine and is essential for cognitive function. Its dysregulation during development can result in lasting neural effects, contributing to ASD-like behaviors [71]. A known inhibitor of AChE that can disrupt neuronal activity is 2,3,7,8-Tetrachlorodibenzo-p-dioxin (TCDD), a dioxin-like compound that suppresses AChE transcription [2]. TCDD exerts its effects by binding to the aryl hydrocarbon receptor (AhR), a ligand-activated transcription factor. Upon activation, the TCDD–AhR complex is brought into the nucleus, where the complex dimerizes with the aryl hydrocarbon receptor nuclear translocator (ARNT) and binds to xenobiotic response elements (XREs) on the DNA. This altered transcriptional regulation downregulates genes such as AChE. In studies using human-derived neurons, the AhR inhibitor CH223191 has been found to reverse TCDD-induced AChE suppression, providing strong evidence for the regulatory role of AhR over AChE.

Several experimental and epidemiological studies further support these mechanistic links, as summarized in Table 1. In neuronal cell culture models, dioxin (TCDD) exposure has been shown to reduce acetylcholinesterase expression through AhR activation [2], while animal studies demonstrate that prenatal or perinatal dioxin exposure disrupts thyroid hormone balance and cerebellar development which are neurodevelopmental alterations consistent with ASD-like outcomes [86]. Notably, the birth cohort found higher autistic trait scores in children with increased dioxin levels in breast milk implicating AhR-mediated disruption of neurodevelopment. Likewise, murine studies indicate that AhR receptor affinity modifies susceptibility to polychlorinated biphenyl (PCB) neurotoxicity, affecting cerebellar and motor pathways relevant to ASD [25]. Together, these findings highlight the translational relevance of environmental pollutant-induced AhR pathway activation in shaping ASD risk.

**Table 1:** Effect of modulators on gene pathway and their association with ASD

Study (Year, Location)	Model/Design	Gene/Pathway	Modulator	Effect on Pathway	ASD-Relevant Outcome (Mechanistic Interpretation)
Ahmed [2**] **(Egypt)	Animal (rat)	AhR → thyroid axis	TCDD	↓ T₃/T₄, ↑ TSH	Disrupted thyroid regulation → abnormal cerebellar maturation and motor delay seen in ASD
Nishijo 2014 (Vietnam)	Cohort (mother–child)	AhR → GABA genes	Dioxin	Altered GABAergic and CaMKII gene expression	Impaired limbic function and socio-emotional behaviors; male-biased vulnerability
Nowack 2015 [88] (Germany)	Birth cohort	AhR	PCDFs, PCBs	Disrupted fetal steroid hormones via androgen/estrogen receptors	Altered empathizing traits and social behavior patterns similar to ASD
Colter 2018 [25] (USA)	Animal (mouse)	AhR + CYP1A2	PCBs	Absence of CYP1A2 ↑ oxidative damage	Motor deficits, nigrostriatal and cerebellar circuit disruption → ASD-like behavior
Glazer 2015 (USA)	Zebrafish embryo	CYP1A1	PCB126	↑ Cyp1a in embryos	Impaired habituation and ↑ anxiety in adults → parallel to ASD sensory dysregulation
El-Ansary et al. [35]	Cross-sectional (human)	CYP1B1 + oxidative markers	Vitamin D status	↓ CYP1B1, ↑ 8-OH-dG, ↑ hs-CRP	Low vitamin D and pollution exposure ↑ neuroinflammation and oxidative stress → greater ASD severity

### Air pollution as a risk factor for ASD

While genetic factors were once presumed to be the primary drivers of ASD, a growing body of research indicates that environmental influences, including exposure to pollutants, play a substantial role in its etiology. The US Environmental Protection Agency has highlighted significant concern in the potential role of air pollution contributing to the etiopathogenesis of ASD. Industrialization, particularly when uncontrolled, has been linked to adverse prenatal and postnatal neurodevelopmental effects, mediated by mechanisms that are still being explored [33]. In a twin study investigating both the genetic heritability of ASD as well as the impact of shared environment, the results revealed that 55% of the risk of developing ASD can be attributed to environmental factors shared between twins [47].

#### Prenatal exposure to airborne pollutants and the increased risk of ASD

Prenatal exposure to air pollutants, including NO2, CO, and PM2.5, has been strongly correlated with an elevated risk of ASD and several other adverse pregnancy outcomes. Overall, a consistent body of epidemiological evidence indicates that higher exposure to ambient air pollution—particularly during pregnancy and the first years of life—is associated with increased odds of ASD in offspring. This association has been observed across multiple pollutants, including fine and coarse particulate matter (PM₂.₅, PM₁₀), nitrogen dioxide (NO₂), ozone (O₃), carbon monoxide (CO), and traffic-related pollutants. However, effect sizes and consistency vary across studies and populations. Meta-analyses and large cohort studies show a modest but heterogeneous association between early life air-pollution exposure and the risk of ASD among children [34], [54]. Table 2 summarizes numerous epidemiological studies examining the association between prenatal and early-life exposure to various air pollutants and ASD risk. During the prenatal stage, the fetus is affected at critical periods of neurodevelopment, with potential long-term effects on brain structure and function. Air pollutants can cross the placenta, leading to inflammation, oxidative stress, and vascular dysfunction. This can lead to placental dysfunction, which in turn affects the nutritional supply to the fetus. In addition, epigenetic changes (i.e., DNA methylation, histone modification) may occur, disrupting gene expression critical for neurodevelopment.

**Table 2:** Association of ASD with prenatal and postnatal exposure to air pollutants and other

**Study** **(Year, Location)**	**Design/Exposure Window**	**Pollutant(s)**	**ASD Assessment Tool**	**Main Findings & ASD-Relevant Outcome **
Talbott [116] (USA)	Case–control; pre-pregnancy → 2 yrs postnatal	PM₂.₅	SCQ > 15 or clinical Dx	45–51% ↑ risk; prenatal + early-life exposure linked to oxidative stress and neuroinflammation during rapid synaptogenesis
Raz 2015 [97] (USA)	Case–control; 9 mo pre- to 9 mo post-birth	PM₂.₅ > PM₁₀	ADI-R, SRS	Highest risk in 3rd trimester when neuronal migration and synaptogenesis peak. Coarse PM showed no effect
Pagalan 2019 [92] (Canada)	Cohort; pregnancy	NO, NO₂, PM₂.₅	ADOS, ADI-R	NO ↑ 7% risk per 10.7 ppb; PM₂.₅ and NO₂ ns. Suggests reactive nitrogen-induced oxidative stress as a mechanism
Chen 2018 [23] (China)	Case–control; first 3 yrs of life	PM₁, PM₂.₅, PM₁₀	DSM-5 clinical dx	Risk ↑ 86%, 78%, 68 for PM₁, PM₂.₅, PM₁₀; linked to systemic inflammation and microglial overactivation during early synaptic pruning
Volk 2013 [125] (USA)	Case–control; pregnancy + 1 st yr	PM₂.₅, PM₁₀, NO₂	ADOS, ADI-R	112–217% ↑ risk; traffic-related PM triggered oxidative stress and BBB disruption in fetal brain
Goodrich 2018 [44] (USA)	Case–control; pregnancy	NO₂, PM₁₀, PM₂.₅	ADOS, ADI-R	High pollution + low folate intake → ↑ ASD risk; folate antioxidant capacity mitigated toxic effect
Kalkbrenner [61] (USA)	Case–control; pre-preg → 1 yr	PM₁₀	DSM-IV-R	36% ↑ risk in 3rd trimester; late-gestation exposure affects synaptic refinement
Windham [132] (USA)	Case–control; prenatal occupational	Exhaust & disinfectants	DSM-IV criteria	Exhaust ↑ 11 ×, disinfectants ↑ 3 × ASD risk; likely via maternal oxidative stress and hormonal disruption
Gong [43] (USA)	Twin cohort; prenatal–9 yrs	PM₁₀, NO	DSM-IV-R	No consistent pattern; possible regional exposure misclassification
Kalkbrenner 2010 (USA)	Case–control; perinatal	Methylene chloride, quinoline, styrene	DSM-IV-R	40–80% ↑ odds; neurotoxic VOC effects through oxidative DNA damage
Roberts 2013 [100] (USA)	Cohort; perinatal	Diesel PM, metals	DSM-IV-R	OR 1.5–2.0; metal and diesel exposure correlated with male-biased susceptibility
Ritz 2018 [99] (Denmark)	Case–control; ± 9 mo preg	PM₂.₅, SO₂, NO₂	ICD-10	SO₂ ↑ 21%, PM₂.₅ ↑ 6%, NO₂ ↑ 8%; urban SO₂ most neurotoxic
Yousefian 2018 [137] (Iran)	Case–control; pregnancy	PM₁₀, SO₂, BTEX	DSM-IV-R	No significant associations; low ambient levels may limit effect
Jung [60] (Asia)	Cohort; 1–4 yrs pre-dx	O₃, CO, NO₂, SO₂	ICD-9	O₃ ↑ 59%, NO₂ ↑ 343%, CO ↑ 37%, SO₂ ↑ 17%; driven by oxidative stress and microglial activation
Jo [59] (USA)	Cohort; pregnancy + 1 st yr	PM₂.₅, PM₁₀, NO₂, O₃	ICD-9	PM₂.₅ ↑ 17% overall, 18% in males; sex-linked oxidative susceptibility
Becerra [11] (USA)	Case–control; pregnancy	O₃, PM₂.₅	DSM-IV-R	12–15% ↑ autism odds; maternal education modified effect
Kaufman 2019 [63] (USA)	Case–control; preg + 1–2 yrs	PM₂.₅ ± O₃	ICD-9	41–84% ↑ risk in early life; postnatal exposure critical to synaptic plasticity
McGuinn 2020 [82] (USA)	Cohort; pregnancy	PM₂.₅	ADOS, ADI-R	↑ PM₂.₅ (5 µg/m^3^) → ↓ adaptive and social skills; supports subclinical neurobehavioral impact

Chemicals. *Abbreviations*: *ADI-R* Autism Diagnostic Interview-Revised, *SRS* Social Responsiveness Scale), *SCQ* Social Communication Questionnaire, *ADOS* Autism Diagnostic Observation Schedule), *ADI-R* Autism Diagnostic Interview-Revised, *DSM-5* Diagnostic and Statistical Manual of Mental Disorders, 5th Edition, *DSM-IV-R* Diagnostic and Statistical Manual of Mental Disorders, 4th Edition, *CATSS* Child and Adolescent Twin Study in Sweden, *ICD-10-CM* International Classification of Diseases, Tenth Revision, Clinical Modification, *ICD-9-CM* International Classification of Diseases, Ninth Revision

A large-scale study in Taiwan (S.-Y. [127] examined trimester-specific associations between air pollutants (CO, NO₂) and ASD risk in over 60,000 newborns. A 1 ppm increase in CO during the first, second, and third trimesters was linked to a nearly doubled ASD risk (HRs: 1.93, (95% CI: 1.55–2.39), 1.77 (95% CI: 1.41–2.22), and 1.75 (95% CI: 1.39–2.21), while a 10-ppb increase in NO₂ corresponded to elevated hazard ratios (1.39 (95% CI: 1.22–1.58), 1.25 (95% CI: 1.10–1.42), and 1.18 (95% CI: 1.03–1.34) across the same trimesters. Numerous studies have demonstrated that exposure to air pollutants can induce oxidative stress, consequently leading to immune dysregulation and neuronal damage [61]. CO, for instance, binds to hemoglobin with a higher affinity than oxygen, causing tissue hypoxia [15]. The greater affinity of fetal hemoglobin for CO than maternal hemoglobin can lead to fetal tissue hypoxia and subsequent reoxygenation [95]. NO2 exposure can generate reactive oxygen species (ROS) in neuronal cells, triggering cell apoptosis [129]. Maternal pollutant exposure can also trigger local inflammation in the fetal brain via placental transfer, amplifying ROS-mediated neuronal damage [4]. Air pollutants can induce epigenetic modifications that disrupt genes that are critical to brain development such as *BDNF, GRIN2B,* and *RELN* [75].

The link between traffic-related pollution and ASD suggests that the cumulative burden of pollutants such as CO, NO2, and PM2.5 during critical windows of neurodevelopment intensifies oxidative stress and neuroinflammation [138]. The convergence of evidence across multiple studies highlights how prenatal exposure to pollutants acts on shared biological pathways rather than independent mechanisms.

Susceptibility windows for ASD have been identified across all three trimesters of pregnancy, with the third trimester posing the highest risk (S.-Y. [127, 130]. ASD can originate as early as the first and second trimesters due to disruptions in cell proliferation and specialization, leading to impaired neural migration, cortical disorganization, delayed neuronal maturation, and altered synapse formation. These developmental disruptions compromise neural network functionality and integration, contributing to ASD pathophysiology [29]. During the third trimester, synapse and neurotransmitter receptor formation coincide with rapid neuronal maturation. PM can cross the placenta, triggering systemic inflammation and oxidative stress. This may reach the developing fetal brain, causing neuroinflammation, neuronal damage, microglial activation, and DNA damage [82]. The converging epidemiological and experimental evidence supports that pollutant exposure during critical developmental windows—particularly late gestation and early infancy—increases ASD risk through biologically plausible pathways. Mechanistically, fine particulate matter and other airborne pollutants can induce systemic inflammation and oxidative stress that cross the placental and blood–brain barriers, triggering microglial activation and neuroinflammation in the developing brain.

Specific interventions have been found to significantly mitigate the effects of pollutants such as O3, NO2, and PM on ASD development. Periconceptional folic acid intake has been shown to reduce the risk of neural tube defects and behavioral problems. A study examining the combined effects of folic acid intake and increased exposure to air pollutants found that participants with lower folic acid intake (≤ 800 μg) and higher air pollutant exposure had a higher risk of ASD, whereas those with higher folic acid intake had a lower risk. This suggests that folic acid intake can counteract the adverse effects of air pollutants by supporting neurogenerative processes [44].

A study on the Danish population analyzed 15,387 children with ASD and 68,139 control subjects to assess the impact of prenatal and early postnatal exposure to environmental pollutants and found that exposure to PM₂.₅ and PM₁₀ during the first nine months of pregnancy correlated more strongly with an increased risk of ASD onset [99]. Providing adequate nutrients and oxygen to the fetus via the placenta promotes normal growth and reduces the risk of low birth weight and intrauterine growth restriction (IUGR) (Van Den [122].

Studies have linked air pollution exposure to preterm birth, likely due to inflammatory responses in the placenta [113]. In the absence of prenatal air pollution exposure, fetal growth proceeds without adverse influences. Without harmful pollutants such as PM2.5, PM10, NO, and O₃, normal placental function is maintained, and the risk of neuroinflammation and neural pathway disruption diminishes. These conditions contribute to improved cognitive, behavioral, and social development [19, 44]. Collectively, these studies indicate that prenatal exposure to common urban air pollutants may contribute substantially to ASD risk, likely through overlapping mechanisms of oxidative stress, inflammation, and disrupted neurodevelopment.

#### Postnatal air pollution exposure and its contribution to ASD development

While prenatal exposure represents a well-established risk window, growing evidence indicates that pollutant exposure during the first years of life may also contribute to ASD development. This period encompasses critical stages of neuronal maturation, synaptogenesis, and myelination, during which the developing brain remains highly vulnerable to environmental insults. Numerous studies have highlighted the crucial role of postnatal exposure to air pollutants in the development of ASD, particularly from the late prenatal third trimester through early infancy [82, 99, 125].

This phase represents a critical developmental window characterized by key processes, including brain structural development, rapid neuronal maturation, and increased myelination and synaptic density [98]. Environmental harms during this phase can dysregulate microglial activation, which is essential for synaptic pruning and proper neuronal circuit refinement. Chronic inflammation, resulting from pollutant exposure, may disrupt microglial function, leading to aberrant synaptic connectivity and neurodevelopmental consequences [82].

Research in China found that exposure to fine particulates (PM1, PM2.5, PM10) during the first three years of life significantly increases the risk of ASD, with the second and third years being the most vulnerable periods [23]. Similarly, studies in the United States consistently link postnatal exposure to air pollution with increased ASD risk, particularly to PM2.5 exposure in the first year of life [63, 125].

In Denmark, a longitudinal cohort study found early infancy exposure to pollutants (SO2, NO2, PM2.5, and PM10) is more strongly associated with ASD than prenatal exposure, highlighting the impact of postnatal environmental factors on neurodevelopment [99].

#### Inconsistent findings and confounding factors

While many studies suggest a correlation between particulate matter exposure and increased ASD risk, some research findings remain contradictory. For example, the Metropolitan Vancouver cohort [92] showed no clear link between prenatal PM2.5 exposure and ASD. In other settings such as in Tehran, Sweden, and Taiwan, studies have also shown inconsistent or non-conclusive results, underscoring the complexity of the air pollution–ASD relationship and the need for further investigation to solidify the correlation. These discrepancies highlight the complexity of disentangling pollution effects, which may depend on exposure assessment methods, population differences, or pollutant mixtures. In addition, dietary factors like maternal folate intake, gut microbiome composition, and the overall nutritional status of an individual can effect the susceptibility to pollutant oxidative stress and act as confounders. Other enviormental factors like heavy metals and pesticides are also very often not accounted for which limits comparibility between findings.

### Biological mechanisms linking air pollution to autism spectrum disorder

#### Inflammation and oxidative stress

**Fig. 2:**
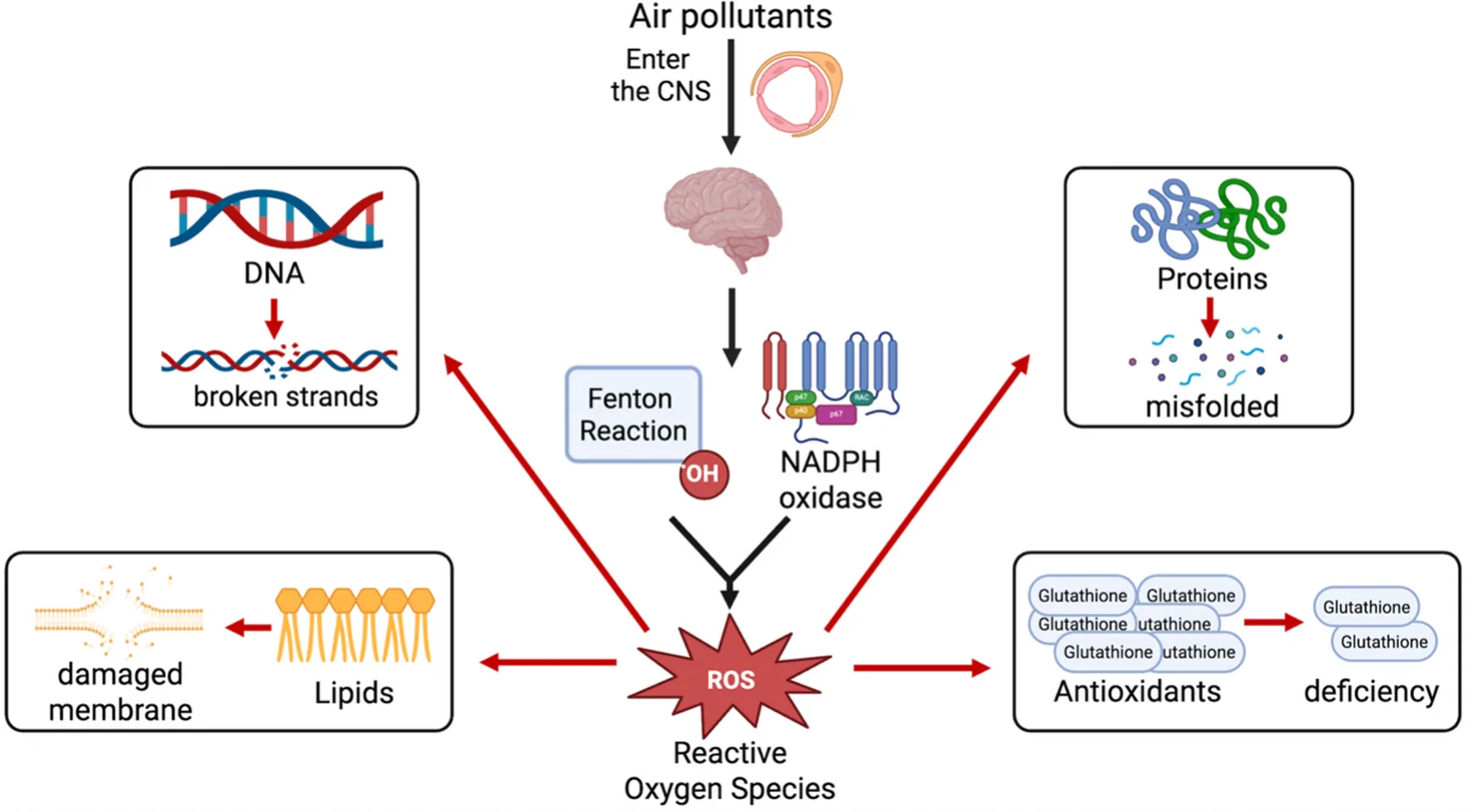
Oxidative stress and air pollution: Reactive Oxygen Species (ROS) as a key mechanism in ASD Pathogenesis. Abbreviations: CNS = central nervous system; ROS = Reactive oxygen species

Once air pollutants enter the CNS, they are recognized by microglia, which respond by producing inflammatory cytokines (IL-1β, TNF-α, and IL-6) (Fig. 2) [28]. Activation of Pattern Recognition Receptors (PRRs) following recognition of air pollutants stimulates intracellular signaling cascades, leading to the induction of nuclear factor kappa B (NF-κB) and the subsequent expression of proinflammatory cytokines [14].

Microglia recognize air pollutants, such as PM2.5, via PRRs, including Toll-like receptors (TLRs), which detect damage-associated molecular patterns (DAMPs) present in these pollutants. These inflammatory responses can impair normal neural development, contributing to an increased incidence of ASD (Fig. 2) [76, 97, 125].

Another critical mechanism by which air pollutants impair neurodevelopment is oxidative stress, a condition characterized by an imbalance between reactive oxygen species (ROS) production and the body's antioxidant defense systems. Pollutants such as PM2.5 can directly or indirectly stimulate ROS production by activating immune cells [58, 65, 121]. ROS are generated via various biochemical mechanisms, including Fenton reactions and NADPH oxidase activity. Fenton reactions occur when transition metals catalyze the conversion of hydrogen peroxide into highly reactive hydroxyl radicals. Specifically, ferrous iron (Fe^2^⁺) reacts with hydrogen peroxide (H₂O₂) to produce hydroxyl radicals (•OH) and ferric iron (Fe^3^⁺) (Fig. 2) [24, 66]. Elevated ROS levels damage cellular structural components, including proteins, lipids, and DNA, thereby impairing cell function and leading to cell death (Fig. 2) [18, 52]. This disruption affects critical neurodevelopmental processes, including neuronal proliferation, migration, and differentiation, leading to brain malformation and dysfunction (Fig. 2) (Chauhan & Chauhan, [21, 125]. On the other hand, NADPH oxidase is an enzyme localized in microglia and neurons and produces superoxide radicals (O₂•⁻) when activated [48]. Exposure to air pollutants like PM can upregulate NADPH oxidase activity which leads to increased superoxide radical production (Fig. 2) [83]. These superoxide radicals can participate in secondary reactions and generate additional ROS. For instance, superoxide radicals can react with hydrogen peroxide in the Haber–Weiss reaction, producing hydroxyl radicals or dismutating to form more hydrogen peroxide, which can then participate in Fenton reactions [24]. The ROS produced through these pathways also activates transcription factors such as NF-κB and AP-1, which amplifies pro-inflammatory cytokine production and exacerbates both inflammation and oxidative damage [22]. This creates a cycle of chronic neuroinflammation and persistent oxidative stress which ultimately contributes to the etiology of neurodevelopmental disorders including an increased incidence of ASD.

#### Disruption of neurodevelopmental processes by airborne pollutants

Air pollution can disrupt brain development through mechanisms including direct neurotoxicity, disruption of endocrine systems, or interference with signaling pathways involved in brain development. Specifically, PM2.5 exposure can affect the expression levels of critical synaptic proteins, such as synaptophysin and PSD-95, which are essential for synaptogenesis and synaptic functionality [73].

**Fig. 3:**
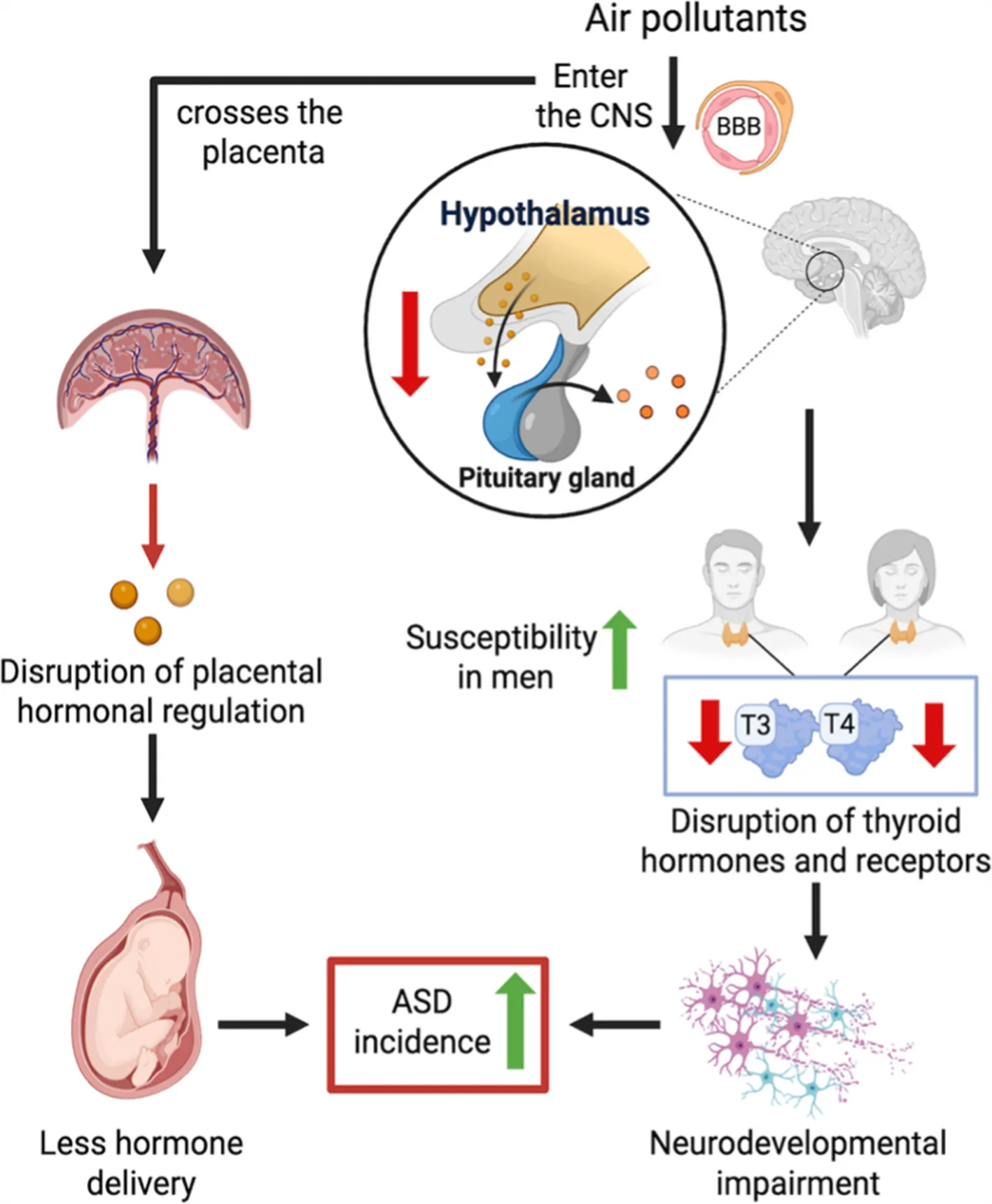
Air pollution, placental hormone disruption, and thyroid dysregulation: a pathway to neurodevelopmental impairment and increased ASD risk. Abbreviations: BBB: Blood Brain Barrier, CNS: central nervous system, ASD: autism spectrum disorder

Many air pollutants disrupt brain development by interfering with the endocrine system by alteration of the balance of hormones which are important for proper neurotransmission and brain maturation (Fig. 3). Thyroid hormones for example regulate genes involved in neuronal differentiation and synapse formation, and they are in particular susceptible to such disruptions. Interference with thyroid hormone receptors at a critical developmental stage can result in structural abnormalities and functional impairments in the brain, increasing the risk of ASD (Fig. 3) [17, 81]. Certain air pollutants act as endocrine disruptors. Particulate matter and dioxin-like compounds can interfere with thyroid hormone signaling, which is critical for fetal brain development,even modest reductions in maternal T4/T3 or receptor-level antagonism during sensitive windows have been associated with cortical abnormalities and cognitive impairments [17, 81]. Experimental data corroborate these effects: perinatal exposure to TCDD (dioxin) in rodent models reduced maternal T4/T3 by up to 75–85% and increased TSH by ~ 300%, producing cerebellar developmental defects in offspring—alterations that parallel neurodevelopmental features implicated in ASD [2]. Figure 3 summarizes how pollutant-induced endocrine disruption converges with neuroimmune and oxidative pathways to impair neurodevelopment.

**Fig. 4:**
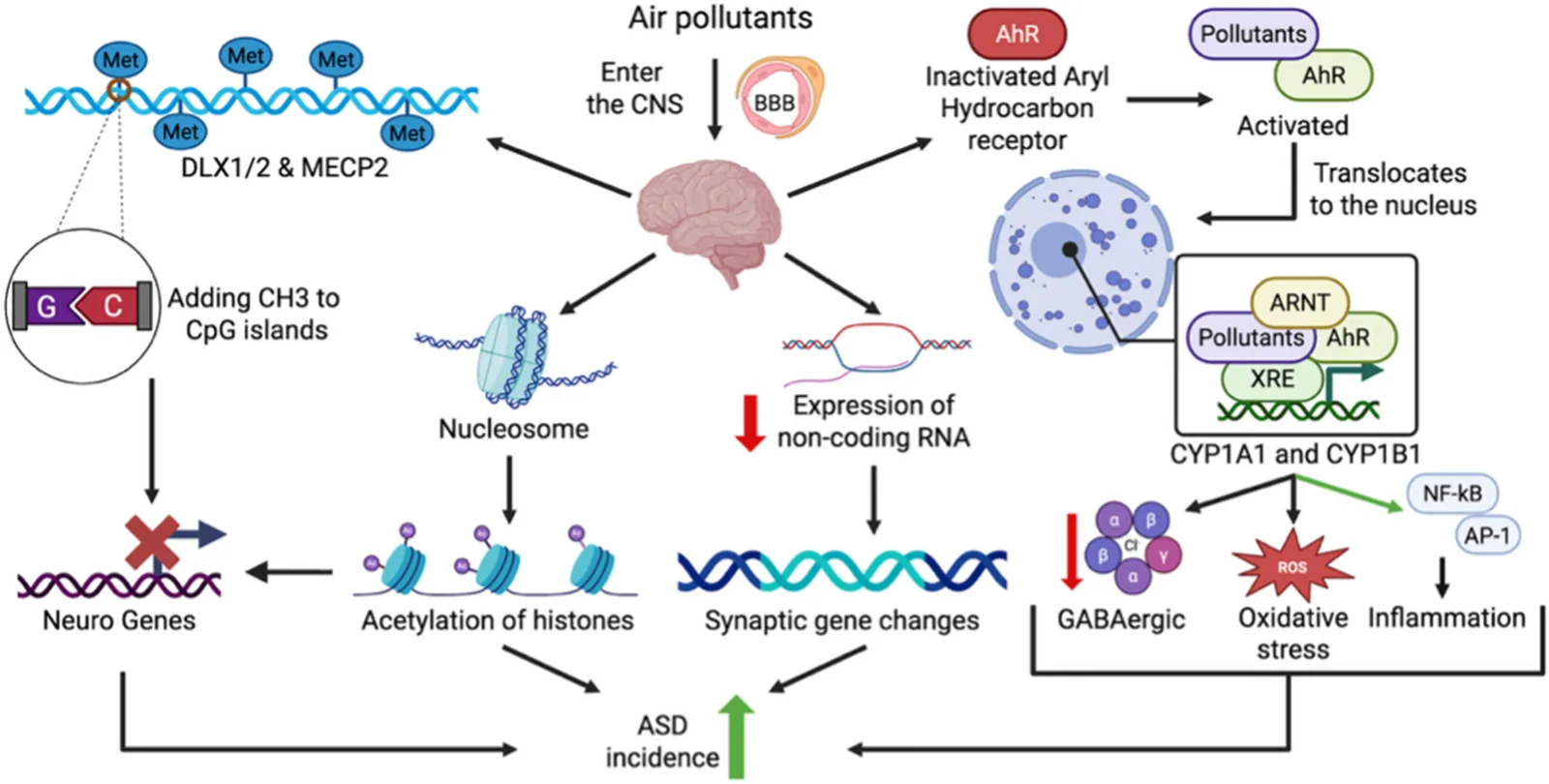
Mechanistic pathways linking air pollution to Autism Spectrum Disorder (ASD): epigenetic modifications, AhR activation, and neurodevelopmental impairment

Persistent organic pollutants, such as Polychlorinated Dibenzo-p-dioxins and Dibenzofurans (PCDD/Fs) and Polychlorinated Biphenyls (PCBs), have also been shown to disrupt critical neurodevelopmental processes by blocking hormone signaling and immune function (Fig. 3). These pollutants bind to the aryl hydrocarbon receptor (AhR), altering endocrine pathways critical to brain development. One of the key focuses of this disruption is the regulation of fetal testosterone, which has a pronounced effect on brain development. This includes how neural circuits essential for social behavior and communication are formed [88]. Moreover, exposure to pollutants such as dioxins has been shown to reduce the expression of essential transcription factors, namely AhR-activated Distal-less Homeobox 1/2 (Dlx1/2), which are involved in the formation of GABAergic interneurons. Less activity of these interneurons, important in correct brain signaling and thinking ability, has been linked to the causation of ASD. This shows that dioxins and PCBs impair brain development largely by derailing hormone signaling and gene expression, resulting in long-term effects on brain development and behavioral outcomes [67, 94]. Figure 4 summarizes these pathways linking air pollution to epigenetic modifications and ASD.

#### Epigenetic modifications induced by air pollution and their impact on ASD

Air pollutants can contribute to epigenetic changes, affecting gene expression and regulatory pathways. The ability of air pollutants to cause these disruptions may depend on their intrinsic oxidative potential (i.e., their capacity to generate ROS and induce oxidative damage). Pollutants with high oxidative potential can trigger oxidative stress, leading to epigenetic modifications such as DNA methylation and histone modifications (Fig. 4) [49]. These alterations result in abnormal gene expression patterns, which may contribute to the neurodevelopmental deficits characteristic of ASD.

PM2.5 can alter DNA methylation patterns, particularly in genes essential for brain development. Pollutant-induced oxidative stress can cause site-specific changes in DNA methylation and histone modifications in genes crucial for neurodevelopment and synaptic signaling. For instance, prenatal exposure to diesel exhaust and its particulate fraction has been associated with methylation alterations in neurodevelopmental genes such as BDNF, MECP2, and RELN, which regulate neuronal growth, plasticity, and cortical organization [13]. Such environmentally driven epigenetic modifications may disrupt transcriptional control during fetal brain development, linking air pollution directly to ASD-related molecular pathways. Studies have identified significant differences in blood DNA methylation patterns between children with ASD and neurotypical controls, suggesting that epigenetically mediated mechanisms contribute to ASD [9].

Prolonged exposure to air pollution is associated with environmentally driven autistic-like characteristics, mediated by epigenetic changes that impair brain structure and function [9]. Prenatal exposure to chemicals (i.e., PCBs and dioxins) can disrupt normal development and neuronal connectivity through gene pathways linked to ASD. These findings highlight the interplay between environmental factors and genetic predispositions in neurodevelopment.

#### Alterations in AhR, CYP1A1, and CYP1B1 pathways due to air pollutants

Dioxins and PAHs are detoxified through mechanisms mediated by the aryl hydrocarbon receptor (AhR). Upon activation by these pollutants, AhR induces the expression of metabolic enzymes, particularly CYP1A1 and CYP1B1, which metabolize dioxins and PAHs. However, the metabolic activity of these enzymes generates reactive oxygen species (ROS) as byproducts, contributing to oxidative stress [45]. This increased oxidative stress can damage cellular components, disrupt signaling pathways, and impair neurodevelopmental processes, potentially increasing the risk of ASD.

The oxidative stress and inflammation triggered by AhR activation and CYP1A/CYP1B induction can lead to epigenetic alterations, including CpG island hypermethylation, histone deacetylation, and microRNA dysregulation. These disruptions interfere with standard gene expression patterns essential for neurodevelopment. Studies in zebrafish and mammalian models have demonstrated that exposure to environmental pollutants that activate AhR can lead to increased expression of CYP1A, behavioral abnormalities, and heightened anxiety [41, 128]***.*** Another study using knockout mouse models exposed to PCBs showed motor dysfunction and increased susceptibility to nigrostriatal dysfunction, a dopamine pathway involved in voluntary movement and cognitive functions, with high-affinity mice showing greater toxicity and brain gene expression changes [25]. In summary, AhR has profound implications for neurodevelopment, and its activation by environmental toxicants initiates a cascade of molecular events, including oxidative stress, DNA damage, and neurotransmitter dysregulation, all of which are implicated in the pathogenesis of ASD.

### Sex differences in the association between air pollution and ASD

The incidence and prevalence of ASD exhibit notable sex distinctions. An epidemiological study shows that ASD occurs at a higher rate in males than in females, with a ratio of 4:1 [30]. Males are more likely to be diagnosed with ASD due to a combination of genetic susceptibility, hormonal variation, and behavioral differences [131].

Research has shown that males may be more vulnerable to the neurotoxic effects of toxins, and this may be the underlying cause of the high prevalence of ASD in males [91]. One explanation for this sex difference is the interaction between maternal immune activation (MIA) and fetal sex hormones: each can alter neurodevelopmental outcomes, and together they may have synergistic effects. Biological vulnerability in males may reflect a stepwise interaction between hormones and immune activation. First, male fetuses exhibit higher prenatal testosterone, which can elevate baseline oxidative stress and modulate neurodevelopmental gene expression [51]. Second, when maternal immune activation (MIA) occurs (e.g., with infection or toxin exposure), the combination of elevated fetal testosterone and MIA potentiates neuroinflammation—manifesting as greater microglial activation and cytokine signaling in vulnerable regions such as the hippocampus and amygdala—thereby increasing the likelihood of atypical circuit development [91, 109]. Third, these hormonally primed, inflammation-driven effects plausibly interact with environmentally induced oxidative stress from air pollutants, compounding risk during sensitive windows of brain development. By contrast, females may be partially protected by lower prenatal testosterone and potentially by X-linked genetic buffering,additionally, sex-specific phenotypes and compensatory behaviors can contribute to underdiagnosis or later diagnosis in females [96]. Some epidemiological studies indeed report sex-specific associations,for instance, fine particulate exposure was linked to ASD risk predominantly in boys, and a case–control analysis found pollutant-related ASD risk was up to 60% higher in males than females. Such findings support the notion of male susceptibility to environmental insults [100]. Further, epigenetic studies reveal heightened DNA methylation anomalies in neurodevelopmental genes in ASD males, elucidating the complex interactions among hormonal, immune, and epigenetic processes that contribute to this sex difference in susceptibility [91].

However, it is essential to note that sex differences in diagnosis may also contribute to the disproportionate sex ratio in ASD. Studies show that females are often undiagnosed or diagnosed later due to differences in ASD clinical presentation. Females with ASD tend to exhibit fewer repetitive behaviors and stronger social skills than males with ASD, leading to misdiagnosis or symptoms being attributed to other conditions (e.g., depression and anxiety). This diagnostic bias likely masks the true female incidence to some extent [96]. This sex-based vulnerability highlights the importance of analyzing ASD risk factors stratified by sex, which is one of the recommendations for future research.

### Air pollution and neurodevelopment: the Middle East and North Africa (MENA) region and beyond

#### Air pollution and ASD in the MENA region

Across the world, the MENA region has significantly higher air pollution levels than other regions. On average, the concentrations of PM₂.₅ were found to be 43.3 µg/m3 in 2019, approximately nine times greater than the World Health Organization’s (WHO) recommended limit of 5 µg/m3 [102]. Between 2021 and 2022 alone, a 13% increase in regional pollution was reported, averaging 3.7 times the WHO guidelines [36]. MENA cities rank among the most polluted globally, with PM₂.₅ levels exceeding the standard threshold. Notably, Baghdad and Cairo have annual PM₂.₅ around 40–46 µg/m3, while Doha and Kuwait City average approximately 35–40 µg/m3 [55]. It is essential to take note of the desert climate of the region in which natural dust contributed 30–60% of the PM in the MENA countries [102]. However, the pollution of the urban areas stems from vehicular exhaust, oil/gas industry, open waste burning, diesel generators, and conflict-related pollution [55]. Ultimately, the worsening air quality over the years has made no city in the MENA region qualified to meet the WHO clean air standards [133]. In light of the established link between air pollution and ASD, examining the prevalence and specific features of ASD in the MENA region, which contains the most polluted cities globally, is a critical research priority.

#### Environmental exposure link to ASD in MENA

The research in the MENA region regarding direct links between environmental exposure and ASD is limited; however, a recent systematic review identified 6 out of 7 Middle Eastern studies demonstrating significant findings regarding the association between ecological pollutants and populations of children with ASD in the region [89]. Several papers focused on the MENA region have studied heavy metals as neurotoxins associated with autistic children populations. In Egypt, children with ASD were found to have significantly elevated levels of lead, mercury, and aluminum correlated with living in factory and traffic-dense areas [136]. Furthermore, an Egyptian case control reported that autistic children had nearly threefold higher hair aluminum levels (20.8 vs. 7.9 μg/g) and significantly elevated mercury concentrations compared to controls [1]. In parallel, similar findings were reported in Oman, where children with ASD had 150–365% higher concentrations of 11 heavy metals in their hair samples relative to neurotypical counterparts [6]. A study in Iraq, a war-affected country, linked neurodevelopmental disorders to exposure to high levels of toxic metals as remnants of military pollution. The study utilized a comparison between Iranian and Iraqi children to highlight that Iraqi children had a markedly higher hair concentration of titanium, with children affected by neurodevelopmental disorders showing further elevations in titanium, lead, arsenic, and cadmium, suggesting increased environmental metal exposure may contribute to neurodevelopmental risk in conflict-affected regions [106]. On the contrary, an Iranian study investigated long-term ambient air pollution exposure among children aged 2–10 years and found no significant association between pollutant exposure levels and increased odds of ASD [137]. Overall, most findings from the MENA region demonstrate an association between environmental toxins and ASD. These further parallel findings discussed above regarding associations between maternal exposure to air pollutants and elevated ASD among offspring in North America and Europe. Although more rigorous research is needed, the MENA region’s high pollution burden can be regarded as a contributing factor to neurodevelopmental harm in the region that extends beyond ASD.

#### Challenges in the MENA region

Several structural barriers hinder research on ASD and air pollution in the MENA region. By 2014, only 26 cities reported their PM₁₀ data to the WHO, highlighting the limited environmental monitoring available in the area and the uncertainty of the available estimates [36]. Furthermore, limited awareness, stigma, and inadequate screening programs are all contributing factors to the underdeveloped infrastructure and ASD surveillance, contributing to the underestimation of ASD [5]. A recent meta-analysis reported a prevalence of approximately 0.14% (1 in 700) of children with ASD in the MENA region, and others reported even lower, despite recent studies from Saudi Arabia and Iraq showing a higher prevalence of 2–6% [5]. The disparities in the results reflect the gaps in ongoing diagnosis and available resources. These restrictions, along with barriers to pollution research, complicate epidemiologic studies of the region and the topic. Thus, improving environmental regulations and ASD screening programs is imperative to strengthening research and protecting child neurodevelopment in the area.

#### Attention deficit hyperactivity disorder (ADHD) and behavioral disorders

In line with findings on air pollution’s association with ASD, there is growing evidence of the same association with elevated prenatal and postnatal exposure to PM₂.₅ and traffic, as well as with a clinical childhood diagnosis of ADHD [139]. Furthermore, a meta-analysis examined this relationship while adjusting for socioeconomic and parental factors, and also found that higher early-life particulate exposures increase the odds of ADHD or ADHD-like behavioral problems [39]. Additionally, a well-known New York-based study also reported an association between polycyclic aromatic hydrocarbons from vehicle exhaust with ADHD symptoms and attention problems at school age [26].

#### Cognitive development and learning disabilities

Alongside ASD and ADHD, air pollution has also been found to impact children’s cognitive function, intelligence, memory, and academic performance. This was evidenced by several cohorts worldwide, which showed that children growing up in highly polluted areas scored lower on cognitive tests [70]. Furthermore, this was quantified in a meta-analysis that showed an average 0.27-point loss in full-scale IQ per 1 µg/m3 increase in PM₂.₅ exposure [8]. This loss is cumulatively significant for cities with high pollution, including the MENA region, where IQ scores could be lowered by several points due to PM₂.₅ exposure [8]. This finding was further supported by UNICEF’s longitudinal study, which found that children exposed to pollutants prenatally had an IQ 4 points lower than their counterparts who were not exposed by age 5 [124].

### Epilepsy and other neurologic outcomes

Although pediatric epilepsy is a neurologic condition not often discussed in association with environmental influences, recent studies have explored the links between them. While research in this field remains relatively new, a 2025 meta-analysis concluded that chronic exposure to PM₂.₅, particulate matter, and NO₂ increases the risk of developing epilepsy among children, while pollutants such as SO₂, CO, and O₃ did not show clear links to epilepsy in that review. The association was found to be small but significant among the six studies analyzed. The results suggest that the neurotoxic impact of air pollutants leads to lowering seizure threshold, contributing to epilepsy. Furthermore, this was supported by reports of increased seizure-related ER visits on days of high particulate or NO₂ levels among children [7]. As research on this topic is still emerging, the impact of air pollution on epilepsy remains incompletely understood, with various findings due to inconsistent study designs and approaches, and is mainly focused outside the MENA region.

In summary, air pollution must be addressed as a serious contributor to cognitive impairment and neurodevelopmental disorders by inducing inflammation, oxidative stress, and structural changes. Thus, reducing exposure to pollution is imperative for pregnant mothers and young children to lower the incidence or severity of long-term neurologic conditions in the MENA region and globally.

### Limitations and future directions

This review highlights progress in understanding the multifactorial etiology of ASD, but also underscores key limitations. The disorder’s heterogeneity and variable diagnostic criteria complicate conclusions on genetic, environmental, and epigenetic interactions.

Observational studies cannot establish causality between air pollution and ASD risk [61, 128]. Variability in study design, population demographics, and methodologies for assessing pollutant exposure limits cross-comparability [92]. Differences in exposure assessment techniques—such as the use of ambient air monitoring, modeled pollutant concentrations, or proxy measures like residential distance from major roads—and the resulting potential for exposure misclassification can also influence findings and reduce comparability across studies. Recent systematic reviews and meta-analyses concur that evidence is accumulating for an association between ambient air pollutants and ASD, while simultaneously emphasizing heterogeneity, potential confounding, and the need for standardized exposure metrics and longitudinal designs. Furthermore, in the MENA region, limited air quality monitoring and weak regulatory enforcement hinder accurate assessment of pollutant exposure, complicating efforts to link environmental data with ASD outcomes. Additionally, underdeveloped ASD surveillance systems, diagnostic gaps, and cultural stigma contribute to underreporting and inconsistent prevalence estimates across the region.

Future research should prioritize longitudinal cohort studies with standardized exposure metrics and leverage multi-omics and machine learning tools to elucidate gene-environment interactions [44, 54]. Machine learning approaches could help identify complex nonlinear interactions between genetic profiles and multiple environmental exposures, potentially uncovering risk patterns not evident from single-factor analyses. Addressing diagnostic biases, particularly in females with ASD, will clarify sex differences in susceptibility and clinical presentation [96]. Research centered on the MENA region should prioritize strengthening air quality monitoring and environmental regulation to improve the accuracy of pollutant exposure assessment. In addition, focusing on enhancing ASD screening programs will allow for more accurate risk assessment and help guide suggested interventions. Investigating therapeutic interventions targeting epigenetic changes and oxidative stress pathways, along with strategies to reduce pollutant exposure, offers promising paths for mitigating ASD risk and advancing personalized medicine.

## Conclusion

ASD is a complex neurodevelopmental condition shaped by an interplay of genetic, biological, immune, and environmental factors. This review highlights the multifactorial nature of ASD, emphasizing the convergence of genetic susceptibility, immune dysregulation, and nutritional influences, with particular focus on pathways involving the aryl hydrocarbon receptor (AhR) and cytochrome P450 enzymes (CYP1A1, CYP1B1). Mounting evidence indicates that exposure to air pollutants significantly increases the risk of ASD. Both prenatal and postnatal exposures may contribute through mechanisms including oxidative stress, inflammation, disrupted neurodevelopment, and epigenetic modification. Moreover, sex-based biological differences appear to modulate vulnerability to pollutant-induced neurotoxicity.

This mechanistic understanding lends biological plausibility to the epidemiological links observed for ADHD, ASD, learning disabilities, and even epilepsy. It also underscores the pressing need for cleaner air as a strategy to safeguard children’s neurological health. Reducing exposure to pollution—especially for pregnant mothers and young children—may help lower the incidence or severity of these lifelong conditions, improving outcomes both regionally and globally.

Collectively, these findings underscore the urgency of mitigating environmental pollution and integrating insights from genetics, epigenetics, and the environment into prevention and therapeutic strategies. Advancing precision approaches that combine molecular, developmental, and ecological perspectives will be essential to reducing ASD risk and improving outcomes for affected individuals.

## Data Availability

No datasets were generated or analysed during the current study.

## Abbreviations

ASD: Autism Spectrum Disorder
PM2.5: Fine Particulate Matter ≤ 2.5 µm
PM10: Coarse Particulate Matter ≤ 10 µm
Nox: Nitrogen Oxides
O3: Ozone
CO: Carbon Monoxide
SO2: Sulfur Dioxide
DPM: Diesel Particulate Matter
CNS: Central Nervous System
CNV: Copy Number Variation
SNP: Single Nucleotide Polymorphism
FMR1: Fragile X Mental Retardation 1 Gene
TSC1/TSC2: Tuberous Sclerosis Complex ½
UBE3A: Ubiquitin Protein Ligase E3A
AhR: Aryl Hydrocarbon Receptor
ARNT: Aryl Hydrocarbon Receptor Nuclear Translocator
XREs: Xenobiotic Response Elements
ROS: Reactive Oxygen Species
PRR: Pattern Recognition Receptor
TLRs: Toll-like Receptors
MZ: Monozygotic
DZ: Dizygotic
GABA: Gamma-Aminobutyric Acid
AchE: Acetylcholinesterase
miRNA: MicroRNA
Dlx1/Dlx2: Distal-less Homeobox ½
PCDD: Polychlorinated Dibenzo-p-Dioxins
PCDF: Polychlorinated Dibenzofurans
DL-PCB: Dioxin-Like Polychlorinated Biphenyls
PCBs: Polychlorinated Biphenyls
TCDD: 2,3,7,8-Tetrachlorodibenzo-p-dioxin
ISR: Integrated Stress Response
OP: Oxidative Potential
CpG: Cytosine-phosphate-Guanine
IUGR: Intrauterine Growth Restriction
ADDM: Autism and Developmental Disabilities Monitoring
